# Creeping Paralysis in a Lady

**Published:** 1886-12-11

**Authors:** J. E. Brown

**Affiliations:** Hon. Sec. "Bardon Grange, Weetwood, near Leeds"


### Difficult Cases.

Creeping Paralysis in a Lady.

The Duchess of Grafton sends us an extract from *Work and Leisure*, which she thinks may possibly give the required information in the case of the poor lady with creeping palsy, mentioned p. 26 of the November number of The Hospital. As there are but few vacancies in the small Home to which the advertisement refers, it may be well to avoid delay in transmiting it. The Duchess knows nothing herself about this Home.

"The Victoria Home for Invalid Ladies.—Permit me to draw the attention of your readers to a small Institution for the benefit of gentlewomen suffering from chronic and incurable complaints: I allude to the Victoria Home for Invalid Ladies, Grosvenor Road, Hyde Park, Leeds, established to provide a permanent and comfortable home, with nursing and medical attendance, for gentlewomen of limited means, who are disqualified for the duties of life by disease, accident, or deformity. The Home is strictly unsectarian, the patients (of whatever denomination) being visited by their own ministers of religion. The house, which is detached and well furnished, stands in its own grounds, and will accommodate eight or ten patients, five of whom are now in, so that early application is desirable. Ladies are admitted by decision of the Committee, and forms of application and terms (which are very moderate) can be obtained from the Lady Superintendent at the Home.

"J. E. Brown, Hon. Sec.

"Bardon Grange, Weetwood, near Leeds."

